# Differential requirements of ciliogenic/ciliopathy module components in restricting Joubert syndrome-associated Arl13b to a *C. elegans* Inv-like ciliary compartment

**DOI:** 10.1186/2046-2530-1-S1-O8

**Published:** 2012-11-16

**Authors:** O Blacque, S Cevik, L Clarke, E Van Wijk, A Sanders, J Van Reeuwijk, K Boldt, M Ueffing, R Roepman, H Kremer

**Affiliations:** 1 University College Dublin, Ireland; 2 Radboud University, Nijmegen, the Netherlands; 3 University of Tubingen, Germany

## 

Cilium dysfunction causes disorders such as Meckel syndrome (MKS), Bardet-Biedl syndrome (BBS), Nephronophthisis (NPHP) and Joubert syndrome (JS). Most ciliopathy proteins localize to cilia where they regulate developmental signaling and transport. Previously we showed that JS-associated Arl13b localises at proximal ciliary membranes, where it regulates transport in *C. elegans*. Now we employ *C. elegans* to investigate the molecular basis of Arl13b ciliary compartmentalisation. First we find that the localisation of *C. elegans* Arl13b (ARL-13) to proximal ciliary regions does not include transition zones. This localisation, reminiscent of the mammalian Inversin (Inv) ciliary compartment, requires an RVxP motif. Using quantitative imaging, ciliopathy and ciliary transport genes were found to differentially restrict ARL-13 to cilia, with IFT, BBS and MKS gene mutants displaying varying ARL-13 accumulations explicitly at periciliary membranes. Specifically, we observed strong accumulations in IFT-B complex mutants, moderate accumulations in IFT-A, BBS and MKS mutants, and no accumulations in NPHP, septin or kinesin-2 mutants. Interestingly, within IFT/BBS/MKS mutant cilia, ARL-13 distributions were unaffected, as was ARL-13 ciliary mobility. Finally, using a TAP-tag immunoprecipitation approach from mammalian HEK293T cells and state-of-the-art mass spectrometry, we identified components of mammalian Arl13b-associated complexes, including the IFT-B subcomplex. These data show that ARL-13 localisation to an Inv-like ciliary compartment requires an RVxP motif and differential contributions by ciliary transport and ciliopathy modules. Our data also suggest that IFT/BBS/MKS modules are not required for ARL-13 distribution and mobility within cilia, but rather its entry into cilia, perhaps by overcoming a transport barrier at the ciliary base.

